# Antiviral Chemotherapy in Avian Medicine—A Review

**DOI:** 10.3390/v16040593

**Published:** 2024-04-12

**Authors:** Ines Szotowska, Aleksandra Ledwoń

**Affiliations:** Department of Pathology and Veterinary Diagnostics, Warsaw University of Life Sciences, 02-776 Warsaw, Poland; aleksandra_ledwon@sggw.edu.pl

**Keywords:** antiviral, birds, avian medicine, viruses, viral infection, therapy

## Abstract

This review article describes the current knowledge about the use of antiviral chemotherapeutics in avian species, such as farm poultry and companion birds. Specific therapeutics are described in alphabetical order including classic antiviral drugs, such as acyclovir, abacavir, adefovir, amantadine, didanosine, entecavir, ganciclovir, interferon, lamivudine, penciclovir, famciclovir, oseltamivir, ribavirin, and zidovudine, repurposed drugs, such as ivermectin and nitazoxanide, which were originally used as antiparasitic drugs, and some others substances showing antiviral activity, such as ampligen, azo derivates, docosanol, fluoroarabinosylpyrimidine nucleosides, and novel peptides. Most of them have only been used for research purposes and are not widely used in clinical practice because of a lack of essential pharmacokinetic and safety data. Suggested future research directions are also highlighted.

## 1. Introduction

Viral diseases are a serious problem in avian species, both poultry and companion birds. Scientists actively look for methods of prevention and eradication of infectious diseases caused by viral pathogens, especially those considered emerging diseases, such as avian influenza, West Nile disease, Newcastle disease, and proventricular dilatation disease (caused by avian bornaviruses). Different approaches focus on immunoprophylaxis, biosecurity, and supportive care, and antiviral therapy is possible. Despite the fact that there is no antiviral drug licensed for use in any avian species, experimental studies mainly include the efficacy of antiviral drugs in specific viral infections and, to a lesser extent, safety profiles of these drugs and pharmacokinetics. Some antivirals licensed for use in human medicine are currently used with the prescribing cascade in therapy for avian viral diseases. Drugs mentioned in the literature to be used in clinical practice include acyclovir in psittacines [1], pigeons [1], backyard poultry [2], and birds of prey [1]. For herpesvirus infections, they include interferon-alpha-2 in psittacines and pigeons for circovirus infections [1], famciclovir in ducklings for duck hepatitis B virus infections [2], and penciclovir in ducks for herpesvirus and duck hepatitis B virus infections [2].

At present, only three antiviral compounds have been licensed for use in veterinary medicine in some countries, including feline interferon-omega (IFN-ω), which is used to reduce mortality and clinical signs of parvovirosis in dogs and to treat cats infected with feline leukemia virus (FeLV) or feline immunodeficiency virus (FIV) in cats [3], and remdesivir and GS-441524, which are both used to treat feline infectious peritonitis (FIP) [4]. There are many local regulations aimed at limiting the use of antiviral drugs, e.g., in the UE, where the use of amantadine, baloxavir marboxil, celgosivir, favipiravir, galidesivir, laktimidomycin, laninamivir, metisazone, molnupiravir, nitazoxanide, oseltamivir, peramivir, ribavirin, rimantadine, tizoxanide, triazavirin, umifenovir, and zanamivir is prohibited in veterinary medicine since 9 February 2023 [5]. To avoid the development of resistance to the antiviral drugs that are listed above, they are reserved for the treatment of certain infections in humans. However, in the 1990s, several reasons for the low use of antiviral chemotherapy in veterinary medicine [6,7,8] were given, including the high cost of development of new substances, particularly for use in animal production, use restricted to a single virus and a specific animal species, difficulties encountered in the development of broad-spectrum antivirals with low cytotoxicity, and a lack of rapid diagnostic techniques, allowing for prompt use of a specific antiviral agent in the course of an acute infection. In avian medicine, two different approaches to antiviral therapy should be considered as diagnostic and therapeutic possibilities, and their aims are different. One is the treatment of species used for food production (mainly poultry) and the second is the treatment of other species kept as pets, such as psittacines and passerines, or for other reasons, like birds of prey. Also, the most commonly diagnosed viral infections vary between different species. Nowadays, drug resistance is becoming a serious threat to public health (noted especially in viruses with zoonotic potential, such as influenza virus) and is monitored by investigators from the whole world because of the ease with which mutations occur in many viruses [9,10,11]. Taking all mentioned above factors into consideration, a review of the literature to assess current knowledge about antiviral chemotherapy in avian species and indicate the possible direction of further research in this field was made.

## 2. Acyclovir

Acycloguanosine (acyclovir [ACV]) is a synthetic acyclic purine nucleoside analog with an antiviral spectrum essentially limited to herpesviruses [12]. Acyclovir triphosphate acts as both a substrate for and an inhibitor of viral DNA polymerase, thus blocking DNA synthesis [13]. The reason for this herpesviruse specificity is that ACV is a substrate for herpesvirus-encoded thymidine kinase, and production occurs in infected cells. Thymidine kinase converts ACV to ACV monophosphate, which is subsequently converted to ACV diphosphate and ACV triphosphate by cellular enzymes. ACV triphosphate is a potent inhibitor of DNA polymerase, as it competes with d-guanosine triphosphate, which is the natural substrate for the viral DNA polymerase. ACV triphosphate has a higher affinity for the enzyme and is preferentially incorporated into the ends of growing DNA chains. Following the incorporation of acyclovir triphosphate into the growing DNA chain, DNA synthesis ceases because ACV lacks the necessary 3′-hydroxyl group to react with incoming nucleotides [12,13,14]. Because ACV triphosphate has a higher affinity for viral polymerase than host cellular polymerases, it has limited toxicity for the host cell. Mutations leading to the development of herpesvirus-resistant strains remain a problem, and ACV does not prevent nor treat latent infections [15].

Most of the avian veterinary interest in ACV as an antiviral agent has been in its use to treat herpesviral infections in different bird species. During an outbreak of Pacheco’s disease, ACV was administered intramuscularly (IM) to potentially infected birds at 25 mg/kg body weight (BW) and was administered in drinking water at 1 g/L and in food (seed mixture) at 400 mg/kg BW for 7 days [16]. Although the majority of treated birds survived, and all untreated birds died; it was not confirmed by any tests that the treated birds were infected by Pacheco’s disease herpesvirus. In monk parakeets (*Myiopsitta monachus*), the pharmacokinetics in healthy specimens and an efficacy in experimentally infected with Pacheco’s disease herpesvirus of ACV administered orally and intramuscularly [17,18]. The oral form of ACV administered by gavage was most effective, and clinical signs and death occurred only after the discontinuation of ACV. At the next step of the experiment, surviving monk parakeets were transferred to cages with seronegative monk parakeets with no known exposure to herpesvirus. There have been no deaths caused by herpesvirus infection in a period exceeding 2 years, which suggests that surviving parakeets did not shed the virus during the time of observation [17]. In a pharmacokinetic study, acyclovir was administered in single doses (20 mg/kg BW intravenously (IV) at 40 mg/kg BW IM and 80 mg/kg BW orally (PO) by crop gavage as a sodium salt for intravenous administration and oral capsule), multiple doses (40 mg/kg BW IM at 8h intervals (q8h) for 7 days, 80 mg/kg BW PO by crop gavage q8h for 4 days), in food (400 mg in 2 qt parrot seed), and water (1 mg of sodium salt for intravenous administration/mL). Results showed that acyclovir reaches therapeutic plasma levels greater than 0.01 pg/mL when administered PO by gavage to the crop, IM, IV, and in the medicated food and water. No evidence of drug accumulation or side effects were noted with any route of administration [18].

Acyclovir was administered towards the end of the outbreak of respiratory disease caused by herpesvirus in a flock of Bourke’s parrots (*Neopsephotus bourkii*), but it was too late for its efficacy to be evaluated [19].

The efficacy of ACV was assessed in pigeons (*Columbia livia*) and budgerigars (*Melopsittacus undulatus*) experimentally infected with pigeon herpesvirus [20]. Intramuscular injections of ACV at 33 mg/kg BW three times daily did not prevent the appearance of clinical disease in infected pigeons and did not reduce viral shedding. The same treatment before infection protected most of the budgerigars for the duration of treatment, but most of them died soon after treatment was stopped. 

The effect of acyclovir on poultry herpesviruses was also investigated [21]. ACV used in doses below 12.5 micrograms/mL proved to be non-toxic for chick embryo fibroblast culture and inhibited in vitro replication of turkey herpesvirus and Marek’s disease virus. It has also been shown to diminish the development of tumors in birds infected with Marek’s disease virus. 

Another study showed that intramuscular administration of ACV at a dose of 10 mg/kg BW q24h prevents the development of clinical signs in chickens experimentally infected with Marek’s disease virus if given for 5 days since day 3 after the challenge, which considerably reduces the mortality rate if given for 9 days starting 14 days after the challenge [22]. 

Plaque reduction assays were used to evaluate possible inhibitory effects of ACV, phosphonoacetate (PAA), and phosphonoformate (PFA) with a plaque-purified isolate of anatid herpesvirus-1 (duck plague virus) [23]. Whereas the interaction between PFA and PAA was additive, synergism occurred with ACV and either PAA or PFA. Drug-resistant mutants of the virus were isolated. 

Results of an ACV pharmacokinetics study in hybrid tragopans (*Tragopan caboti x Tragopan temminckii*) where ACV was administered to five healthy adult birds, three males and two females, in a single dose of 40, 80, or 120 mg/kg BW PO suggest that a dosage of 120 mg/kg BW PO q12h in tragopans may achieve effective plasma concentrations (1.0 µg/mL) for potential treatment and prevention of herpesviral infections [24]. Although the nonlinearity of acyclovir pharmacokinetics in this species makes dose extrapolation difficult, this suggestion is based on maintaining plasma acyclovir concentrations above the accepted effective acyclovir plasma level (1.0 µg/mL) for 12 h after administration of the 120 mg/kg dose. Throughout all treatments, all birds appeared clinically healthy based on clinical examination and hematologic testing. 

## 3. Abacavir and Its Derivates

Abacavir and its derivates are widely used in human medicine, especially HIV type 1, in combination with other antiviral drugs to enhance efficacy against HIV [25,26]. Because of low solubility, high cytotoxicity, low bioavailability, and a lack of target specificity of many antiviral chemotherapeutics [27], phosphorylation was proposed to increase the bioavailability and decrease the cytotoxicity of the activated nucleoside analogs in virus-infected cells [28]. As these phosphorylated nucleosides have high permeability and bioavailability, the focus is on producing nucleotide prodrugs, which finally release parent nucleosides at a specific site [25,29,30,31]. The mechanism proposed for abacavir [32,33,34] involves its conversion by cytosolic enzymes to carbovir monophosphate (CBV-MP), which is subsequently phosphorylated by host cellular kinases to carbovir triphosphate (CBVTP). Triphosphate is a potent and selective inhibitor of HIV reverse transcriptase by competing with dGTP for incorporation into viral DNA [32]. 

There is evidence that abacavir and its derivates showed antiviral activity against Newcastle disease virus (NDV), which is the cause of Newcastle disease (ND) [25,35]. Due to its high mortality and morbidity, NDV is a devastating virus and causes huge economic loss to the poultry industry. One study showed that abacavir and its phosphorylated compounds exhibit binding affinity to a fusion protein of Newcastle disease virus, suggesting that these compounds can be considered potent anti-NDV compounds or NDV inhibitors [25]. Because of the better binding affinity of three compounds, these three compounds were chosen for the synthesis of phosphorylated compounds and further in vitro studies on DF-1 cells. One of them (ABC-1) showed marked anti-NDV activity compared to two other compounds and has been further studied [25,35]. The antiviral activity of ABC-1 was also better than the parent compound, abacavir, which has been evidenced by its significant decrease in HA titer and lipid peroxidation and increase in antioxidant enzyme levels. Further studies of ABC-1 showed that tissue oxidative stress has been reduced and the expression of fusion protein caused by NDV infection has been inhibited in the NDV-infected chickens treated with ABC-1 in a dose of 2 mg/kg BW PO from the fourth day post-infection [35]. Immunolocalization, PCR, and flow cytometry analysis also showed that the novel phosphorylated compounds are effective in inhibiting the fusion protein expression, which is important in the replication of NDV.

## 4. Adefovir and Its Derivates

Adefovir and its derivates, such as adefovir dipivoxil, are antiviral drugs used in human medicine to treat HIV, hepatitis B, and cytomegalovirus infection [36]. The expression of the antiviral efficacy of adefovir requires phosphorylation to the active adefovir diphosphate moiety [37,38]. The diphosphate competitively inhibits deoxyadenosine triphosphate as a substrate for reverse transcriptase [39,40] and/or causes chain termination when incorporated into the growing DNA chain [37].

Duck hepatitis B virus (DHBV) is a model for research on the treatment of hepatitis B virus because of a similar characteristic of both viruses [41,42]. Research on adefovir antiviral activity against DHBV was performed when given as a single drug [43,44,45] or in combination with other therapeutics [46] and vaccines [47]. Both studies conducted by Heijtink et al. [43,44] showed that adefovir and its derivates have antiviral activity against DHBV both in vitro (in DHBV-infected duck hepatocytes) and in vivo given to Pekin ducks at a dose of 30 mg/kg BW q24h. Another study [45] showed that adefovir at a dosage of 15 mg/kg BW q24h has potent activity against DHBV without any hepatological, hematological, or biochemical evidence of systemic toxicity at this dose. However, a rebound of viral replication was observed after drug withdrawal when adefovir was given as a single drug [43,44,45,46,47]. It has been confirmed that at clinically achievable concentrations, the antiviral effects of all two-drug combinations containing adefovir, penciclovir, and lamivudine against DHBV in vitro are additive or synergistic [46]. Also, the anti-DHBV effect of combinations containing all three drugs is approximately additive, as measured by the inhibition of intracellular DHBV replication, although this is not consistently reflected by comparable inhibition of virus-specific protein synthesis. Results obtained by Guerhier et al. [47] suggest the presence of an additive effect of adefovir and DNA vaccine and a sustained decrease in intrahepatic DHBV DNA observed 12 weeks after the end of therapy.

## 5. Amantadine

Amantadine is a water-soluble tricyclic amine used as an antiviral agent for the prophylaxis and treatment of influenza A virus in human medicine [48]. Amantadines’ antiviral mechanism of action is based on interference with the release of infectious viral nucleic acid into the host cell through interaction with the transmembrane domain of the M2 protein of the influenza A virus [49]. Amantadine by an indirect dopamine-releasing action and direct stimulation of dopamine receptors is also widely used in the treatment of Parkinson’s disease [50]. 

In veterinary medicine, amantadine is used mainly to reduce nociception associated with chronic pain [51,52]. The use of amantadine in antiviral therapy in veterinary medicine is not common, but the irresponsible use of amantadine in commercial poultry in China [53] and Egypt [54] allowed for the emergence of drug-resistant mutants among lethal influenza strains, rendering this drug ineffective for treating human infections [55,56]. Analysis of global H5N1 sequencing data (isolated from both human and avian hosts) showed that the frequency of L26I/V27A mutation in M2, which is the response to resistance to amantadine, is linearly correlated with the mortality rates of human H5N1 infections [55]. Also, analysis of avian influenza isolates from poultry in Vietnam [10], Southeast Asia, and North America [57] showed that resistance to amantadine is a real problem. Results of a study conducted by scientists at the Friedrich–Loeffler–Institut in Germany indicated that the resistance to amantadine conferred by the presence of the amantadine resistance marker at position 31 (Ser31Asn) of the M2 protein evolved rapidly after the application of amantadine in commercial poultry [58]. Asn31 increased virus entry into the cells and cell-to-cell spread and was genetically stable for several passages in cell culture. The co-infection of cell culture with resistant and sensitive strains resulted in the dominance of resistant strains over sensitive viruses, even in the absence of selection by amantadine. Researchers concluded that the rapid emergence, stability, and domination of amantadine-resistant variants over sensitive strains limit the efficacy of amantadine in poultry.

However, some attempts to use amantadine in poultry [56,58,59,60,61,62,63] and pet birds were made [64,65,66].

The first study of the prophylactic use of amantadine in poultry was described in 1970 [59]. The optimum prophylactic dosage regimen included the administration of amantadine (10 mg/kg BW orally once daily or 0.025–0.05% amantadine incorporated into feed pellets) at least 2 days pre-infection to 23 days post-infection in turkeys experimentally infected with HPAIV H5N9.

A comprehensive study performed in the 1980s showed that both amantadine and rimantadine administered in water at a concentration of 0.01% were well accepted by chickens, were characterized by good pharmacokinetic properties, and prevented infection with H5N2 influenza virus when given concomitantly with the inoculation with the virus [60]. Further parts of this study demonstrated that amantadine administered in water at a concentration of 0.01% is effective in the treatment of chickens experimentally infected with the virulent H5N2 influenza virus, but it did not prevent virus shedding. Moreover, the majority of contact birds (which were also given amantadine 0.01% in water) died, and the virus recovered from these birds was resistant to amantadine in the plaque assay. The last experiment showed that the vaccine administered at the time of contact did not reduce mortality in the contact birds, but when vaccine and amantadine treatment were administered simultaneously, none of the contact birds died and they all developed antibodies. 

Another study also showed rapid development of resistance to amantadine in the H5N2 avian influenza virus during an experiment that simulated layer flock treatment [61].

A few studies showed that amantadine-resistant strains of the avian influenza virus were irreversible, stable, and transmissible with pathogenic potential comparable to the wild-type virus and, even more, amantadine-resistant strains replaced the wild-type virus and became dominant [56,62,63].

Based on anecdotal reports [64], a group of clinically healthy, seropositive, avian bornavirus-shedding African gray parrots (*Psittacus erithacus*) were treated with amantadine for 6 weeks, with no apparent effect on fecal viral shedding [65]. 

A pharmacokinetic study showed that once-daily oral administration of amantadine at 5 mg/kg BW to orange-winged amazon parrots (*Amazona amazonica*) maintained plasma concentrations above those considered to be therapeutic for chronic pain in dogs without any observed adverse effects [66].

## 6. Ampligen

Ampligen is an immunomodulator and interferon inducer, which in one study was used alone and in combination with ganciclovir and coumermycin A1 to treat ducks congenitally infected with duck hepatitis B virus [67]. When used alone, ampligen decreased the amount of serum and liver viral DNA. In combination with ganciclovir, the antiviral effect was additive with a greater inhibition of viral DNA replication within the liver. The combination of ampligen with coumermycin A1 also resulted in the inhibition of viral replication but to a lesser extent than ampligen alone. When all three agents were used together, viral DNA replication was again inhibited. During all used treatment regimens, the level of circulating duck hepatitis B surface antigen (DHBsAg) measured in serum remained unchanged. At the end of the treatment period for all regimes, analysis of viral DNA forms in the liver showed that the viral relaxed circular and supercoiled DNA forms had persisted. Within 1 week of cessation of therapy, viral replication had returned to levels recorded before treatment. Interferon-like activity was detected in the sera of the majority of the treated ducks during ampligen therapy. 

## 7. Azo Derivates

Azo dyes (-N=N-) are a class of compounds that are used as dyes and pigments and biomedical applications [68]. Azo derivates show antiviral [69,70,71,72], antibacterial [73], antifungal [74], antitumor [75], hypotensive, anti-inflammatory, and antioxidant effects [76]. 

In one study, embryonated eggs were inoculated with a Newcastle disease virus and an avian influenza virus [77]. The results of the hemagglutination (HA) test in the case of anti-NDV and anti-AIV potential of azo compounds revealed that three of five tested azo compounds actively inhibited NDV growth at a concentration of 0.1 mg/100 μL, and two of five compounds were able to inhibit AIV replication.

## 8. Baloxavir Marboxil

Baloxavir marboxil (BXM) is a prodrug of baloxavir acid used to treat influenza in human medicine [78]. Baloxavir acid inhibits cap-dependent endonuclease, an essential protein involved in the initiation of viral transcription by cleaving capped mRNA bound to basic polymerase 2. 

BXM shows antiviral activity against H5 highly pathogenic avian influenza strains [9,79]. In chickens infected with the H5N6 HPAI virus, a single administration of 2.5 mg/kg BW of BXM immediately after inoculation was determined as the minimum dose required to fully protect chickens from the HPAI virus [80]. The concentration of baloxavir acid, the active form of BXM, in chicken blood at this dose was sufficient for a 48 h antiviral effect post-administration. In a further experiment, when BXM was given at a dose of 20 mg/kg BW q12h PO for 5 days from the moment when clinical signs were noticed at 24 h post-infection to mimic the field situation of an HPAI outbreak, all eight treated birds died. One chicken survived up to 6 days post-infection.

## 9. Didanosine

Didanosine (2,3-dideoxyinosine, DDI) is a dideoxy analog of purine nucleoside inosine that is a reverse transcriptase enzyme inhibitor and inhibits the replication of HIV [81]. Didanosine is metabolized intracellularly by a sequence of cellular enzymes to its active part dideoxyadenosine triphosphate, which inhibits reverse transcriptase competitively [82]. Phosphorylated didanosine derivates are designed [29,31,83] to overcome the disadvantages of didanosine, such as a short plasma half-life, low bioavailability, and dose-dependent cellular toxicities; it is unstable in its acidic conditions [84] and has poor permeability through intestinal epithelium and low oral bioavailability [85]. In one study, phosphorylated didanosine derivate DDI-10 was chosen from a series of designed derivates and showed antiviral activity against NDV in vitro, as evidenced by a significant reduction in plaque formation and cytopathic effects [86]. 

In vivo, in NDV-infected chicken, it was shown that superoxide dismutase and catalase were significantly raised, and lipid peroxidation and HA titer levels were decreased upon treatment with 1.5 mg/kg BW of DDI-10 compared to 3 mg/kg BW of DDI [86]. Histopathological alterations in NDV-infected tissues were restored in chickens treated with DDI-10. Another study showed that in NDV-infected chickens treated with DDI-10 glutathione-dependent enzymes, GPx, GST, and GR significantly increased and oxidation and nitration levels decreased compared to NDV-infected chickens [87].

## 10. Docosanol

*n*-Docosanol is a long-chain (C-22) saturated fatty alcohol with antiviral activity against herpes simplex virus (HSV), cytomegalovirus, human immunodeficiency virus (HIV), respiratory syncytial virus (RSV), influenza A virus, and murine Friend leukemia virus [88,89]. Some studies showed that *n*-docosanol has no direct antiviral effect [90], but a metabolic intracellular conversion prevents virus entry and, consequently, allows the cells to abort the infectious cycle of many enveloped viruses [88,91]. 

*n*-Doconasol given for 4 successive days at a dose of 40 and 60 mg/kg BW showed therapeutic activity against velogenic Newcastle disease virus (NDV) in domestic chickens [92]. NDV shedding was also reduced in treated birds. 

## 11. Entecavir

Entecavir (ETV) is a deoxyguanosine analog with activity against hepatitis B virus (HBV) [93]. ETV inhibits reverse transcription, DNA replication, and transcription in the viral replication process [94]. 

Studies showed that ETV is a potent, safe, rapid-acting, and long-term suppressor of DHBV replication but is not able to eliminate the virus from infected duck organisms [94]. The treatment protocol consists of a DNA vaccine given 50 days post-infection, and ETV treatment that started 14 days post-infection at a dose of 0.1 mg/kg BW/day for 244 days did not result in any additional reduction in viral load compared to ETV used alone [95]. A higher dose (1 mg/kg/day) of ETV and different vaccination protocols lead to the clearance of DHBV-infected hepatocytes in ~50% of ETV-treated ducks [96]. In this treatment regimen, ducks were given ETV for 14 days starting from the day when the animals were inoculated and vaccinated at day “0”, day 7, and day 14 post-infection. 

## 12. Fluoroarabinosylpyrimidine Nucleosides

Three fluoronucleosides, 1-(2-fluro-2-deoxy-13-D-arabinofuranosyl)-5-iodocytosine (FIAC), 1-(2-fluoro-2-deoxy- [3-D-arabinofuranosyl)-5-iodouracil (FIAU), and its thymine analog (FMAU) have been shown to have potent anti-herpes simplex virus activity in vitro and in vivo [97,98,99,100,101].

It was demonstrated that all three fluoroarabinosylpyrimidine nucleosides (FIAC, FIAU, and FMAU) were potent inhibitors of MDV and HVT in chick kidney cells (CKCs), but only two of them (FMAU and FIAU) were active against these viruses in chick embryo fibroblasts (CEFs) and splenic lymphocyte cultures [102].

## 13. Ganciclovir

Ganciclovir (9-[(1,3-dihydroxy-2-propoxy)methyl]guanine) is a guanosine analog that is a potent inhibitor of viruses of the family herpesviridae [103] and hepadnaviridae [104]. The primary mechanism of action is inhibition of the replication of viral DNA by ganciclovir-5′-triphosphate (ganciclovir-TP) by chain termination. This inhibition includes a selective and potent inhibition of the viral DNA polymerase [103]. 

Ganciclovir was tested on a duck hepatitis B infection model to assess its safety and efficacy when given alone [105,106,107,108,109] and with nalidixic acid [109]. In a study conducted by Wang et al. [105], 5-week-old ducks congenitally infected with DHBV were treated with 21-day courses of twice-daily intraperitoneal injections of ganciclovir in a dose of 10 mg/kg BW q24h (alone or with pre-treatment with prednisolone 1 mg/kg once daily IM followed by a 10 mg/kg BW q24h 21-day ganciclovir course) or 30 mg/kg BW q24h without noticing any side effects in clinical observation and laboratory markers of liver, renal, and hematological function. In both protocols with only ganciclovir, the level of viremia rebounded to greater than pre-treatment levels within 2 weeks of cessation of therapy. This rebound phenomenon was not observed in follow-up samples obtained from ducks treated with prednisolone and ganciclovir. In the cited study, ganciclovir treatment failed to produce any consistent or significant reduction in the level of circulating duck hepatitis B surface antigen (DHBsAg). In other studies, when ganciclovir was given at a dose of 10 mg/kg BW q24h for 3 [106] and 4 weeks [107], similar observations were noted. After cessation of treatment, viremia returned to detectable levels within 4 days, despite that the treatment resulted in a substantial reduction in both viremia and liver DHBV DNA replicative intermediates. Also, long-term therapy of congenitally DHBV-infected ducks with ganciclovir at a dose of 10 mg/kg BW q24h for 24 weeks failed to permanently stop DHBV replication [108]. A combination of ganciclovir at a dose of 10 mg/kg BW q24h with nalidixic acid at a dose of 250 mg/kg BW q12h resulted in a substantial decrease in viremia, with DHBV DNA levels becoming undetectable [109]. However, during the follow-up period, the DHBV DNA in serum returned to detectable levels. A pharmacokinetic study showed that ketoprofen given at a dose of 2 mg/kg BW IV at the same time as ganciclovir at a dose of 10 mg/kg BW IV caused increased plasma concentration and prolonged the elimination half-life of ganciclovir in chukar partridges (*Alectoris chukar*) [110]. 

## 14. Interferon

Interferons (IFNs) are a class of cytokines elicited on challenge to the host defense and are essential for mobilizing immune responses to pathogens [111]. Divided into three classes, type I, type II, and type III, IFNs do not have direct antiviral activity but affect cells, inducing the synthesis of antiviral agents. Interferons activate a number of enzymes in the cell that allow the degradation of viral genetic material, causing the so-called antiviral status [112]. In human medicine, IFNs have been widely used in the treatment of many diseases, such as hepatitis B, hepatitis C, and multiple sclerosis [112]. IFNs also show anti-cancer effects. In general, the interferon system is species-specific—it will protect cells against viruses only in the same or closely related animal species from which it originates (e.g., interferon produced in rabbit cells will not protect mouse cells, but human interferon can protect monkey cells from some viral attack) [113].

In avian species, the cross-species antiviral activity of goose interferon-gamma against duck plague virus in vitro in duck embryo fibroblasts (DEFs) has been confirmed [114]. Recombinant duck interferon-alpha (rDuIFN-α) inhibits avian influenza (AIV) H5N1 virus in vitro and in vivo [115]. It was demonstrated that the IM administration of 1 × 10^5^ U rDuIFN-α significantly reduces the morbidity and mortality of H5N1 AIV infection in Pekin ducks.

There was an attempt to treat circovirus infection in gray parrots (*Psittacus erithacus*) with type 1 interferon-alpha of feline origin or avian gamma interferon derived from poultry cell cultures [116]. In the feline-origin interferon-treated group, there was no improvement in clinical observations and hematological parameters (severe leukemia) in all 12 birds, which died or were euthanized within 30 weeks. Avian gamma-interferon seemed to be effective in the treatment of psittacine beak and feather disease caused by circovirus in gray parrots. In the avian gamma-interferon-treated group, seven of ten birds were alive after 30 weeks and exhibited normal total white blood cell counts, and samples of blood and feather pulp taken in week 30 were negative for circovirus by PCR. 

It has been demonstrated that the combination of ribavirin with recombinant interferon-alpha shows a synergistic effect against avian bornaviruses in vitro [117].

A comprehensive study focused on the effects of nitric oxide (NO) and chicken interferon-gamma on the replication of MDV showed that the addition of S-nitroso-N-acetylpenicillamine resulted in the production of NO and reduced replication of MDV and turkey herpesvirus (HVT) in a dose-dependent manner in vitro in chicken embryo fibroblasts [118]. Further experiments demonstrated that recombinant chicken interferon-gamma and lipopolysaccharide (LPS) inhibited MDV replication in chicken embryo fibroblasts. The addition of NG-monomethyl L-arginine (NMMA) by blocking the production of NO reversed the inhibition of viral replication. Additionally, LPS and interferon were tested when used alone; LPS did not inhibit MDV replication, and recombinant chicken interferon-gamma caused a nonsignificant inhibition of viral replication.

A few studies demonstrated that chicken interferon-alpha showed an inhibitory effect on avian influenza viruses in vitro [119], in ovo [120], and in vivo [120,121,122].

## 15. Ivermectin

Recently, drug repurposing became an option for the treatment of emerging diseases, as developing new effective antiviral drugs against a particular pathogen is very time-consuming, laborious, and expensive. Ivermectin, an antiparasitic drug, is one of the recently tested drugs in Flaviviridae [123,124] and SARS-COV-2 [125,126,127,128] treatment. 

It was shown that ivermectin is a potent inhibitor of Usutu virus (USUV) replication in vitro [124]. USUV is a mosquito-borne arbovirus within the genus Flavivirus and family Flaviviridae. Similar to the closely related West Nile virus (WNV), USUV infections are capable of causing mass mortality in wild and captive birds, especially blackbirds, and both viruses are able to infect humans [129]. In the last few years, a massive spread of USUV has been noticed in the avian populations, mainly the common blackbird (*Turdus merula*) in Germany and other European countries [130]. Also, some anecdotal reports indicated improved clinical outcomes in cases of West Nile virus and USUV infections in hawks and owls treated with ivermectin [131].

## 16. Novel Peptides

Peptides with antiviral activity characterize biocompatibility, specificity, and effectiveness, and they also overcome the limitations of existing drugs [132]. Synthetic peptides are designed using virtual screening technology and molecular docking technology, which shorten the research cycle and costs and significantly increase the possibility of screening for desirable results [133,134]. 

Because hydropericardium hepatitis syndrome (HHS) caused by fowl adenovirus serotype 4 (FAdV-4) is the cause of significant economic losses to the poultry industry [135,136], research on treatment possibilities was conducted [137]. Treatment with one (P15) of eight investigated peptides significantly inhibited virus proliferation in vitro in LMH cells, probably through the binding of the peptide to the C-terminal knob domain of the FAdV-4 Fiber2 protein. 

Another study showed that four peptides derived from the Marek disease virus (MDV) glycoprotein gH (gHH1, gHH2, gHH3, and gHH5) and one peptide derived from MDV glycoprotein gB (gBH1) have potent antiviral activity against MDV both in vitro in plaque formation assays in primary chicken embryo fibroblast cells (CEFs) and in vivo in lesion formation assays on a chorioallantoic membrane (CAM) [138].

## 17. Lamivudine

Lamivudine is a nucleoside derivative antiviral drug with a competitive inhibitory effect on viral DNA synthesis and extension [139]. It has been shown to inhibit reverse transcriptase and, therefore, inhibits the replication of human immunodeficiency virus (HIV) by competition binding with cellular nucleotides both in vitro and in vivo [140,141,142,143,144].

As a type of retrovirus, reverse transcriptase also plays a key role in the life cycle of avian leukosis virus subgroup J (ALV-J). The efficacy of lamivudine against ALV-J was tested in vivo in chickens [145]. It has been shown that lamivudine could inhibit ALV-J replication by competing with normal nucleotides for reverse transcriptase binding and inhibit cDNA transcription and extension with a mechanism similar to that of HIV inhibition, but it cannot kill the virus completely.

Lamivudine was also confirmed to show an antihepadnaviral effect in primary duck hepatocytes infected with DHBV in vitro [146]. It has been also shown that the antiviral activity of lamivudine in combination with adefovir [45] or penciclovir [45,146] against DHBV in vitro is additive or synergistic. 

## 18. Nitazoxanide

Originally developed and commercialized as an antiprotozoal agent, nitazoxanide was later identified as a broad-spectrum antiviral drug [147,148]. Nitazoxanide and its active metabolite tizoxanide show antiviral activity in vitro against influenza A and B [147], hepatitis B virus [149], hepatitis C virus [149], paramyxovirus [150], yellow fever virus [151], HIV [152], and many different mammalian coronaviruses [153].

The efficacy of nitazoxanide against West Nile virus in vitro and in vivo has been demonstrated [154]. Treatment of two-week-old chickens resulted in a significant reduction in NDV replication in the trachea and lungs at 72 h post-infection.

## 19. Oseltamivir

Oseltamivir is the oral prodrug of GS4071, a selective inhibitor of influenza A and B viral neuraminidase widely used in human medicine [155].

Oseltamivir given at a dose of 120 mg/kg BW q12h from 1 day before inoculation to 7 days post-infection could not prevent HPAI infection of inoculated chickens, but treatment limited morbidity, mortality, and chicken-to-chicken transmission of HPAI virus infection [156]. Another study confirmed that oseltamivir reduces the replication of low pathogenic AIV, significantly in chickens and completely in domestic ducks [157]. Research on the safety of oseltamivir in chicken embryos demonstrated that it is non-toxic at concentrations of 0.1, 1.0, and 10.0 mg/kg BW [158].

None of the few mentioned studies conducted to evaluate the efficacy of oseltamivir in avian species reported the emergence of resistant strains. In contrast to amantadine, oseltamivir-resistant H5N1 avian influenza viruses isolated from domestic and wild birds probably emerged due to spontaneous mutations rather than exposure to oseltamivir [159,160,161,162].

## 20. Penciclovir and Famciclovir

Famciclovir (FCV) is the oral form of the potent and selective antiherpesviral and antihepadnaviral agent penciclovir (PCV) and was developed because of the poor absorption of penciclovir given orally [163,164,165]. Similarly to acyclovir, guanosine analog penciclovir is a highly selective inhibitor of herpesviruses DNA polymerase because it is phosphorylated and thereby only activated in herpesvirus-infected cells [163,164,165,166,167].

In avian species, antiviral activity against duck hepatitis B virus both in vitro [168] and in vivo [169,170,171,172,173] has been mainly studied. An in vitro study compared the antihepadnavirus activities of penciclovir and ganciclovirin primary duck hepatocyte cultures congenitally infected with the duck hepatitis B virus (DHBV) [168]. Both compounds inhibited DHBV DNA replication to a comparable extent during continuous short-term treatment, but penciclovir was more active both during longer-term continuous treatment and in washout experiments. Based on anti-DHBV activity, it has been demonstrated that young ducklings efficiently metabolize FCV given orally to the parent compound, PCV, which is activated to exert an antiviral effect not only in the liver but also in the nonhepatic tissues (kidney, pancreas, and spleen) [169]. In different studies [170,171], it was demonstrated that penciclovir given to DHBV-infected ducks in different treatment protocols (dose ranges from 10 mg/kg BW q24h for 4 weeks to 20 mg/kg BW q12h and 100 mg/kg BW q12h for 3 weeks) suppress viral replication, but it is not able to completely kill the virus, and DHBV DNA plasma levels increased after the cessation of treatment, which indicated that virus replication had resumed. The same effect has been noticed when giving famciclovir at a dose of 5 mg/kg BW and 25 mg/kg BW q12h for 3 weeks [171]. Long-term therapy of congenitally DHBV-infected ducks with penciclovir at a dose of 10 mg/kg BW q24h for 12 or 24 weeks resulted in similar effects—viral replication was inhibited but during the follow-up period, DHBV DNA returned to pre-treatment levels [172]. Nicoll et al. [173] found that penciclovir given to congenitally DHBV-infected ducklings at a dose of 10 mg/kg BW q24h for 17 days controlled viral replication and reduced viral burden in hepatocytes but not in the bile duct epithelial cells, which might be an important reservoir of the virus that is relatively unaffected by antiviral treatment and may play a role in relapse after cessation of therapy. In all mentioned in vivo studies [169,170,171,172,173], no side effects were observed during and after PCV or ACV therapy.

## 21. Ribavirin

Ribavirin is a broad-spectrum antiviral drug that is a purine analog and can potentially act on numerous steps of the virus life cycle, such as the inhibition of translation due to a reduction in cellular GTP pools or incorporation as a cap analog, which inhibits translation, the inhibition of genome or transcript capping by suppression of GTP synthesis or direct competition, the inhibition of RNA synthesis directly via active-site binding or a reduction in GTP synthesis, ambiguous incorporation into RNA causing increased mutation and production of non-viable genomes, enhancement of the antiviral immune response, and the prevention of spread and pathogenesis [174]. In human medicine, ribavirin is used mainly to treat hepatitis C virus infections, but there is evidence of a benefit in RSV infection and Lassa fever [174].

Ribavirin toxicity and efficacy against Newcastle disease virus in ovo has been studied [175]. Inoculation of a 0.1 mL solution with the lowest used concentration of 10 μg/mL did not stop the replication of the virus, while inoculation of a 0.1 mL solution with the highest used concentration of 40 μg/mL was toxic, and embryos in eggs died. Inoculation of a 0.1 mL solution with a concentration of 20 μg/mL was non-toxic and had antiviral activity. 

Ribavirin was proven to inhibit the replication of parrot bornaviruses in vitro but was not able to eliminate the virus [176,177]. Ribavirin showed synergistic effects against parrot bornaviruses with recombinant IFN-α in avian cells [122]. Avian bornaviruses were discovered in 2008 in parrots suffering from proventricular dilatation disease (PDD), which is a chronic neurologic and gastrointestinal disorder of birds belonging to the order Psittaciformes [178,179]. Recently, evidence that avian bornaviruses also cause the disease in canaries (*Serinus canaria*) was published [180,181]. Bornaviruses that are able to infect other species of birds, such as ducks, geese, swans, and estrildid finches, were also described [182]. However, their pathogenicity is still unclear. Because avian bornaviruses are a common cause of losses in psittacine collections and can be a threat to endangered species conservation programs, they are currently one of the most intensively studied exotic bird viruses in the field of treatment and prevention [182].

## 22. Zidovudine

Zidovudine is an antiviral drug used in HIV infection treatment in human medicine [183]. Mechanism studies show that zidovudine is phosphorylated by the host cell enzymes. The triphosphate of zidovudine appears to be the active form of the drug and it competes well with thymidine 5′-triphosphate for binding to the HIV reverse transcriptase and also functions as an alternative substrate. The incorporation of zidovudine monophosphate results in chain termination.

Avian leukosis virus (ALV) is an oncogenic virus belonging to alpha retrovirus [184], which is the cause of avian leukosis in poultry [185]. ALV also cause immunosuppression in infected birds, resulting in huge economic losses due to secondary infections [186,187]. One study [188] showed that a combination of zidovudine and short hairpin RNA was able to significantly inhibit ALV subtype J replication in vitro.

## 23. Conclusions

The main conclusion is that the current knowledge about the use of antiviral drugs in avian species in clinical practice is poor. However, numerous experimental studies showed that many antiviral drugs can effectively inhibit the replication of some avian viruses, depending on the substance and virus species. There are many more in vitro studies than in vivo experiments. Even promising results of these in vitro studies should be confirmed in experimentally infected birds if they have not been performed yet. In some studies, even though they were conducted on birds, the main goal was to determine drug efficacy on animal models to assess its usefulness in human medicine (e.g., ducks infected with duck hepatitis B virus, which are animal models of hepatitis B virus infection in human), not to determine if the drug can be useful in the treatment of disease in birds. There are a few pharmacokinetic studies of specific antiviral substances in avian species. A small number of pharmacokinetic studies makes it difficult to determine dosage and use them in clinical practice. Dose extrapolation from mammals to avian species is hard because of many differences in metabolism (e.g., metabolic rate and specific metabolic processes). 

Considering antiviral therapy in birds, the risk of drug-resistance development should also be kept in mind, especially in zoonotic infections, such as HPAI. Antiviral therapy with classic antiviral drugs in poultry is generally useless because of residues of drugs in tissues and eggs, and as mentioned above, a high risk of drug-resistant viral strain development. However, further research on the use of antiviral drugs in birds is warranted because, in some situations, antiviral therapy can be the only way to save endangered bird species. Also, some viral infections in birds kept in a zoo and companion animals could be treated.

Future studies in this field should focus on pharmacokinetics in drugs proven to be effective in specific infections in birds in vitro and in vivo to determine dosage and enable their wider application in clinical practice. Further studies on substances that were not tested in avian viral infections yet are also warranted.

## Data Availability

Not applicable.
